# Relationship between long non-coding RNA and prognosis of patients with coronary heart disease after percutaneous coronary intervention: A protocol for systematic review and meta-analysis

**DOI:** 10.1097/MD.0000000000023525

**Published:** 2020-12-18

**Authors:** Fei Wang, Xiaoqing Cai, Piqi Jiao, Yan Liu, Bin Yuan, Peng Zhang, Hongbin Liu, Ling Ma

**Affiliations:** Department of Cardiovascular Medicine, 940th Hospital of PLA Joint Service Support Force, Lanzhou, Gansu, China.

**Keywords:** coronary heart disease, long non-coding RNA, meta-analysis, percutaneous coronary intervention, prognosis, protocol

## Abstract

**Background:**

Long non-coding RNA (lncRNA) can predict the prognosis of patients with coronary heart disease (CHD) after obtaining percutaneous coronary intervention (PCI), while this conclusion still needs to be further confirmed. Therefore, this study attempted to explore the relationship between lncRNA and prognosis in CHD patients after PCI.

**Methods:**

The database was retrieved from China National Knowledge Infrastructure (CNKI), Chinese Biomedical literature Database (CBM), Chinese Scientific and Journal Database (VIP), Wan Fang database, PubMed, and EMBASE. Hazard ratios (HRs) and its 95% confidence interval (CIs) were applied to assess the prognostic effects of lncRNA on overall survival (OS). RevMan 5.3 and STATA 16.0 software were used to perform meta-analysis.

**Results:**

The results of this meta-analysis would be submitted to peer-reviewed journals for publication.

**Conclusion:**

This review provided a comprehensive overview of the relationship between lncRNA and prognosis in CHD patients after PCI, and offered recommendations for clinical practices or guidelines.

## 1 Introduction

Mainly caused by atherosclerosis,^[1]^ coronary heart disease (CHD) is one of the leading causes of death in the world.^[2]^ Clinical manifestations of CHD include arrhythmia, angina pectoris, myocardial infarction, heart failure, and sudden death.^[3]^ Vascular smooth muscle cell changes phenotype after injury, migrates to intima and attracts extracellular matrix polymerization, thus playing an important role in the development of atherosclerosis.^[4]^ Hyperlipidemia, hypertension, and smoking belong to the risk factors of CHD.^[5–7]^ The prognosis of CHD patients is regulated by many factors such as angina pectoris, myocardial infarction, ischemic heart failure, and sudden death.^[8]^

Revascularization is the most effective method for the treatment of coronary heart disease. Although it cannot completely cure coronary heart disease, it can greatly improve CHD patients’ clinical symptoms, thus increasing their life quality and survival rate. At present, coronary artery reconstruction mainly involves coronary artery bypass grafting and percutaneous coronary intervention (PCI).^[9]^ With the emergence of PCI, its rapid development and the continuous emergence of new anticoagulant and antiplatelet drugs have constantly broken through the limited field of PCI therapy.^[10]^ However, no effective biological index can be adopted to judge the prognosis of CHD after PCI.

With the production and rapid development of the second generation sequencing, especially the application of RNA sequencing, long non-coding RNA (lncRNA) has gradually become a hot spot in the field of life science. The expression level of lncRNA in blood samples may be regarded as a prognostic and diagnostic biomarker. In order to analyze the effect of LncRNA on the prognosis of CHD after PCI more accurately, this study reviewed the literature on the relationship between lncRNA and prognosis in CHD patients after PCI, and applied meta-analysis to evaluate the relationship between lncRNA and prognosis in patients CHD after PCI.

## 2 Methods

### 2.1 Study registration

This meta-analysis protocol is based on the Preferred Reporting Items for Systematic Reviews and meta-analysis Protocols (PRISMA-P) statement guidelines. The protocol of the systematic review was registered on Open Science Framework, and the registration number is DOI 10.17605/OSF.IO/RAZ6J.

### 2.2 Literature retrieval

We searched China National Knowledge Infrastructure (CNKI), Chinese Biomedical literature Database (CBM), Chinese Scientific and Journal Database (VIP), Wan Fang database, PubMed, EMBASE, and all these electronic databases, without language restrictions. The PubMed search strategy was illustrated in details in Table 1, and other electronic databases adopted similar search strategies.

**Table 1:** Search strategy (PubMed).

Number	Search terms
1	Coronary Disease [MeSH]
2	Coronary Heart Disease [Title/Abstract]
3	CHD[Title/Abstract]
4	Coronary Diseases[Title/Abstract]
5	Coronary Heart Diseases[Title/Abstract]
6	Disease, Coronary[Title/Abstract]
7	Disease, Coronary Heart[Title/Abstract]
8	Diseases, Coronary[Title/Abstract]
9	Diseases, Coronary Heart[Title/Abstract]
10	Heart Disease, Coronary[Title/Abstract]
11	Heart Diseases, Coronary[Title/Abstract]
12	Coronary Artery Disease[MeSH]
13	Arteriosclerosis, Coronary[Title/Abstract]
14	Atherosclerosis, Coronary[Title/Abstract]
15	Coronary Arteriosclerosis[Title/Abstract]
16	Coronary Atherosclerosis[Title/Abstract]
17	Arterioscleroses, Coronary[Title/Abstract]
18	Artery Disease, Coronary[Title/Abstract]
19	Artery Diseases, Coronary[Title/Abstract]
20	Atheroscleroses, Coronary[Title/Abstract]
21	Coronary Arterioscleroses[Title/Abstract]
22	Coronary Artery Diseases[Title/Abstract]
23	Coronary Atheroscleroses[Title/Abstract]
24	Disease, Coronary Artery[Title/Abstract]
25	Diseases, Coronary Artery[Title/Abstract]
26	OR/1-25
27	RNA, Long Untranslated[MeSH]
28	LINC RNA[Title/Abstract]
29	LincRNAs[Title/Abstract]
30	Long Intergenic Non-Protein Coding RNA[Title/Abstract]
31	Long Non-Coding RNA[Title/Abstract]
32	Long Non-Protein-Coding RNA[Title/Abstract]
33	Long Noncoding RNA[Title/Abstract]
34	Long ncRNA[Title/Abstract]
35	Long ncRNAs[Title/Abstract]
36	RNA, Long Non-Translated[Title/Abstract]
37	Long Intergenic Non Protein Coding RNA[Title/Abstract]
38	Long Non Coding RNA[Title/Abstract]
39	Long Non Protein Coding RNA[Title/Abstract]
40	Long Non-Translated RNA[Title/Abstract]
41	Long Untranslated RNA[Title/Abstract]
42	Non-Coding RNA, Long[Title/Abstract]
43	Non-Protein-Coding RNA, Long[Title/Abstract]
44	Non-Translated RNA, Long[Title/Abstract]
45	Noncoding RNA, Long[Title/Abstract]
46	RNA, Long Non Translated[Title/Abstract]
47	RNA, Long Non-Coding[Title/Abstract]
48	RNA, Long Non-Protein-Coding[Title/Abstract]
49	RNA, Long Noncoding[Title/Abstract]
50	Untranslated RNA, Long[Title/Abstract]
51	ncRNA, Long[Title/Abstract]
52	ncRNAs, Long[Title/Abstract]
53	OR/27–52
54	Percutaneous Coronary Intervention[MeSH]
55	Percutaneous Coronary Revascularization[Title/Abstract]
56	Coronary Intervention, Percutaneous[Title/Abstract]
57	Coronary Interventions, Percutaneous[Title/Abstract]
58	Coronary Revascularization, Percutaneous[Title/Abstract]
59	Coronary Revascularizations, Percutaneous[Title/Abstract]
60	Intervention, Percutaneous Coronary[Title/Abstract]
61	Interventions, Percutaneous Coronary[Title/Abstract]
62	Percutaneous Coronary Interventions[Title/Abstract]
63	Percutaneous Coronary Revascularizations[Title/Abstract]
64	Revascularization, Percutaneous Coronary[Title/Abstract]
65	Revascularizations, Percutaneous Coronary[Title/Abstract]
66	OR/54–65
67	Prognos^∗^[Title/Abstract]
68	Survival[Title/Abstract]
69	OR/67–68
70	26 AND 53 AND 66 AND 69

### 2.3 Inclusion criteria for study selection

(1) CHD was diagnosed based the coronary artery score of the American College of Cardiology.^[11]^
(2) LncRNA was expressed in serum or plasma.
(3) Reported LncRNA survival-related data.
(4) Patients were divided into LncRNA positive and LncRNA negative.
(5) Published as full-text articles.

Papers without sufficient data, literature reviews, case series, conference summaries, animal studies, and other unrelated studies were excluded from the analysis.

### 2.4 Data collection and analysis

#### 2.4.1 Selection of studies

**Figure 1:**
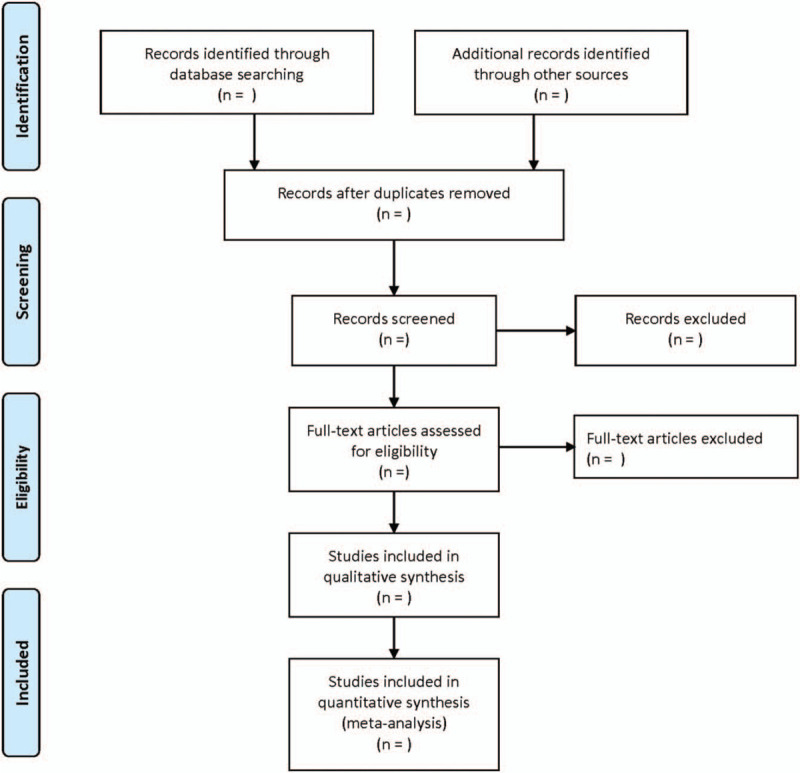
Flow diagram showing literature filtration process.

First of all, 2 researchers read and checked out the titles and abstracts of relevant literatures independently. After excluding the literatures that obviously do not meet the inclusion criteria, and downloading the remaining literatures for further full-text reading, the literatures that really meet the inclusion criteria were enrolled. The 2 researchers cross-checked the results. A self-made data extraction form was made to extract the required information from the included literatures. If there are differences, a third party would involve the discussion or consultation in this study. The flowchart of the study selection (Fig. 1) could provide a detailed description.

#### 2.4.2 Data extraction

The extracted information includes the title of the paper, name of the first author, journal, year of publication, country, race, age, sex, sample size, LncRNA detection method, LncRNA type, overall survival (OS), hazard ratio (HRs), and 95% confidence interval (CIs). If we can’t access survival data directly, we would obtain HRs and 95% CIs from the Kaplan–Meier survival curves by referring to Engauge Digitizer version 4.1 (http://digitizer.sourceforge.net/).

### 2.5 Literature quality evaluation

As a bias risk assessment tool for observational studies recommended by Cochrane Collaboration, Newcastle-Ottawa quality Assessment scale (NOS) was adopted to evaluate the quality of the included studies.^[12]^ Any dispute was settled by discussing. NOS consists of the following 3 quality parameters: selection, comparability, and result evaluation. According to these parameters,^[13]^ each study was scored from 0 to 9.

### 2.6 Measures of prognosis

OS was taken as prognostic outcomes, and the results were expressed as HRs with 95% CIs.

### 2.7 Management of missing data

If there exists insufficient or missing data in the literature, we would only analyze the currently available data and discuss its potential value.

### 2.8 Statistical analysis

Statistical analysis was performed with STATA 14.0 (STATA Corporation, College Station, TX) and RevMan 5.3 (The Nordic Cochrane Centre, The Cochrane Collaboration, 2014). The 95% CIs and HRs were utilized to evaluate the relationship among LncRNA expression and OS. First of all, chi-squared test and *I*^*2*^ test were used to test the heterogeneity of the included study (test level α = 0.1). *I*^2^ ≤ 25% indicated that the heterogeneity was small, 25% < *I*^2^ ≤ 50% marked moderate heterogeneity, and *I*^2^ > 50% revealed that there was a high degree of heterogeneity among the research results. When there is no heterogeneity among the research results, the fixed effect model is adopted for meta-analysis. When there exists heterogeneity among the research results, the stability of the conclusion is verified, and the random effect model is decided for meta-analysis.

### 2.9 Additional analysis

#### 2.9.1 Subgroup analysis

According to the detection methods of LncRNA, ethnicity, the source of survival data, and type of LncRNA, we analyzed the subgroup.

#### 2.9.2 Sensitivity analysis

Sensitivity analysis was conducted to assess the impact of individual studies on the overall merger value.

#### 2.9.3 Reporting bias

If the number of studies included in a certain outcome index was no <10, funnel chart could be used to evaluate publication bias.^[14,15]^ Besides, Egger and Begg test were adopted for the evaluation of potential publication bias.

### 2.10 Ethics

Our research data were derived from published literatures, because there was no patient recruitment and personal information collection. Therefore, ethical approval was not required.

## 3 Discussion

Cardiovascular disease, especially CHD, is still the leading cause of death worldwide, resulting in a significant social and economic burden. Although drug therapy, PCI, and other treatments can improve the prognosis of CHD, the mortality rate is still very high.^[16]^ Therefore, it is necessary to detect CHD in the early stage, especially before the occurrence of left ventricular dysfunction. It is very helpful to use circulatory or imaging biomarkers to identify the high risk of adverse cardiovascular events in CHD patients.^[17]^ However, the lack of available CHD biomarkers limits the risk prediction.^[18]^

In the past 10 years, the research on lncRNA in terms of the occurrence and development of CHD and other heart diseases has become more and more in-depth. LncRNA not only regulates cell proliferation, apoptosis, injury, autophagy, and differentiation, but also participates in the occurrence and development of a variety of cardiovascular diseases through different molecular mechanism. Therefore, it can be regarded as a new regulatory factor for diagnosis and treatment.

Some plasma LncRNA, such as ANRIL, LincRNA-p21, and myocardial infarction-related transcription factor-1, significantly increases in atherosclerosis and may play an important role in the pathogenesis of atherosclerosis.^[19–22]^ Pezawas et al^[23]^ proposed that the expression of LncRNA UCA1 in plasma of patients with acute myocardial infarction decreasing significantly in the early stage, and gradually rising after the third day, so it was regarded as a marker of acute myocardial infarction. Hu and Hu^[24]^ put forward that ANRIL has good diagnostic value for CHD, and its high expression is correlated with the increased degree of stenosis, increased inflammation and poor OS in CHD patients. LncRNA is stable in plasma and other body fluids, so it can be applied as a biomarker for some diseases. We hope to conduct a meta-analysis in accordance with PRASMA statement to provide more evidence for LncRNA as a prognostic marker of CHD after PCI. However, this study also has some limitations. Our search does not include learning languages other than Chinese and English, so it may lead to some selective bias. Most importantly, there may be some heterogeneity due to the application of different treatments in various studies.

## Author contributions

**Conceptualization:** Ling Ma, Fei Wang.

**Data curation:** Xiaoqing Cai, Peng Zhang.

**Resources:** Piqi Jiao.

**Software:** Yan Liu, Bin Yuan, Hongbin Liu.

**Writing – original draft:** Ling Ma, Fei Wang.

**Writing – review & editing:** Ling Ma, Fei Wang.

## References

[1] Wang XB Han YD Sabina S HDAC9 Variant Rs2107595 modifies susceptibility to coronary artery disease and the severity of coronary atherosclerosis in a Chinese Han population. PLoS One 2016;11:e0160449. 27494404 10.1371/journal.pone.0160449 PMC4975504

[2] Dai X Wiernek S Evans JP Genetics of coronary artery disease and myocardial infarction. World J Cardiol 2016;8:1–23. 26839654 10.4330/wjc.v8.i1.1 PMC4728103

[3] Zhang Y Huang J Yang X Altered Expression of TXNIP in the peripheral leukocytes of patients with coronary atherosclerotic heart disease. Medicine (Baltimore) 2017;96:e9108. 29245343 10.1097/MD.0000000000009108 PMC5728958

[4] Gao S Wassler M Zhang L MicroRNA-133a regulates insulin-like growth factor-1 receptor expression and vascular smooth muscle cell proliferation in murine atherosclerosis. Atherosclerosis 2014;232:171–9. 24401233 10.1016/j.atherosclerosis.2013.11.029 PMC4334121

[5] De Bacquer D van de Luitgaarden IAT De Smedt D Socioeconomic characteristics of patients with coronary heart disease in relation to their cardiovascular risk profile. Heart 2020;0:1–8. 10.1136/heartjnl-2020-317549 33067329

[6] Krittanawong C Kumar A Wang Z Coronary artery disease in the young in the US population-based cohort. Am J Cardiovasc Dis 2020;10:189–94. 32923100 PMC7486526

[7] Irawati S Wasir R Floriaan Schmidt A Long-term incidence and risk factors of cardiovascular events in Asian populations: systematic review and meta-analysis of population-based cohort studies. Curr Med Res Opin 2019;35:291–9. 29920124 10.1080/03007995.2018.1491149

[8] Harada M Miura T Kobayashi T Clinical impact of complete revascularization in elderly patients with multi-vessel coronary artery disease undergoing percutaneous coronary intervention: a sub-analysis of the SHINANO registry. Int J Cardiol 2017;230:413–9. 28040276 10.1016/j.ijcard.2016.12.093

[9] Kiaii B Teefy P Hybrid coronary artery revascularization: a review and current evidence. Innovations (Phila) 2019;14:394–404. 31500492 10.1177/1556984519872998

[10] Changjiang H Jian Q Yuan Z Tirofiban combined with fondaparinux for post-pci treatment of patients with acute coronary syndrome and mild renal insufficiency. Cell Biochem Biophys 2015;73:603–7. 27259300 10.1007/s12013-015-0580-1

[11] Fihn TD Gardin JM Abrams J 2012 ACCF/AHA/ACP/AATS/PCNA/SCAI/STS guideline for the diagnosis and management of patients with stable ischemic heart disease: a report of the American College of Cardiology Foundation/American Heart Association Task Force on Practice Guidelines, and the American College of Physicians, American Association for Thoracic Surgery, Preventive Cardiovascular Nurses Association, Society for Cardiovascular Angiography and Interventions, and Society of Thoracic Surgeon. J Am Coll Cardiol 2012;126:3097–137. 10.1016/j.jacc.2012.07.013 23182125

[12] Stang A Critical evaluation of the Newcastle-Ottawa scale for the assessment of the quality of nonrandomized studies in meta-analyses. Eur J Epidemiol 2010;25:603–5. 20652370 10.1007/s10654-010-9491-z

[13] Zhang Q Jin Y Li X Plasminogen activator inhibitor-1 (PAI-1) 4G/5G promoter polymorphisms and risk of venous thromboembolism - a meta-analysis and systematic review. Vasa 2020;49:141–6. 31920171 10.1024/0301-1526/a000839

[14] Lewis SJ Zammit S Gunnell D Bias in meta-analysis detected by a simple, graphical test. BMJ Clin Res 1997;315:629–34. 10.1136/bmj.315.7109.629 PMC2127453 9310563

[15] Duval S Tweedie R Trim and fill: a simple funnel-plot-based method of testing and adjusting for publication bias in meta-analysis. Biometrics 2000;56:455–63. 10877304 10.1111/j.0006-341x.2000.00455.x

[16] Daneault B Généreux P Kirtane AJ Comparison of Three-year outcomes after primary percutaneous coronary intervention in patients with left ventricular ejection fraction <40% versus ≥40% (from the HORIZONS-AMI trial). Am J Cardiol 2013;111:12–20. 23040595 10.1016/j.amjcard.2012.08.040

[17] Malaud E Merle D Piquer D Local carotid atherosclerotic plaque proteins for the identification of circulating biomarkers in coronary patients. Atherosclerosis 2014;233:551–8. 24530963 10.1016/j.atherosclerosis.2013.12.019

[18] Wykrzykowska JJ Garcia-Garcia HM Goedhart D Differential protein biomarker expression and their time-course in patients with a spectrum of stable and unstable coronary syndromes in the Integrated Biomarker and Imaging Study-1 (IBIS-1). Int J Cardiol 2011;149:10–6. 20060182 10.1016/j.ijcard.2009.11.033

[19] Hung J Miscianinov V Sluimer JC Targeting non-coding RNA in vascular biology and disease. Front Physiol 2018;9:1655. 30524312 10.3389/fphys.2018.01655 PMC6262071

[20] Shi X Wei YT Li H Long non-coding RNA H19 in atherosclerosis: what role? Mol Med 2020;26:72. 32698876 10.1186/s10020-020-00196-w PMC7374855

[21] Zhang JR Sun HJ LncRNAs and circular RNAs as endothelial cell messengers in hypertension: mechanism insights and therapeutic potential. Mol Biol Rep 2020;47:5535–47. 32567025 10.1007/s11033-020-05601-5

[22] Zhao Z Sun W Guo Z Mechanisms of lncRNA/microRNA interactions in angiogenesis. Life Sci 2020;254:116900. 31786194 10.1016/j.lfs.2019.116900

[23] Pezawas T Diedrich A Robertson D Risk of arrhythmic death in ischemic heart disease: a prospective, controlled, observer-blind risk stratification over 10 years. Eur J Clin Investig 2017;47:231–40. 28102901 10.1111/eci.12729 PMC5392777

[24] Hu Y Hu J Diagnostic value of circulating lncRNA ANRIL and its correlation with coronary artery disease parameters. Braz J Med Biol Res 2019;52:e8309. 31411246 10.1590/1414-431X20198309 PMC6694403

